# A seroprevalence survey of anti-SARS-CoV-2 antibodies among individuals 18 years of age or older living in a vulnerable region of the city of São Paulo, Brazil

**DOI:** 10.1371/journal.pone.0255412

**Published:** 2021-07-29

**Authors:** João Luiz Miraglia, Camila Nascimento Monteiro, Alexandre Giannecchini Romagnolo, Rafaela Xavier Gomes, Cristóvão Pitangueiras Mangueira, Eliane Aparecida Rosseto-Welter, Juliana Gabriel Souza, Marina da Gloria dos Santos, Ranier Nogueira dos Santos, Karina I. Carvalho, Daiana Bonfim

**Affiliations:** 1 Diretoria de Atenção Primária e Rede Assistencial, Hospital Israelita Albert Einstein, São Paulo, São Paulo, Brasil; 2 Instituto Israelita de Ensino e Pesquisa Albert Einstein, Hospital Israelita Albert Einstein, São Paulo, São Paulo, Brasil; 3 Medicina Diagnóstica e Ambulatorial, Hospital Israelita Albert Einstein, São Paulo, São Paulo, Brasil; 4 Department of Nutrition, Case Western Reserve University, Cleveland, Ohio, United States of America; Universidade Federal de Sao Paulo, BRAZIL

## Abstract

A second wave of COVID-19 has demonstrated how challenging it will be to achieve sustained control of the disease, even with vaccination underway in many countries. Therefore, it remains relevant to keep improving our understanding of the distribution of COVID-19, especially of asymptomatic individuals, among different populations, and particularly in vulnerable regions. Hence, this population-based serosurvey had the objective of estimating the prevalence of individuals 18 years of age or older infected by SARS-CoV-2, and the proportion of asymptomatic individuals, among a vulnerable population living in an urban setting. This was a cross-sectional single-stage cluster sampling serosurvey conducted between September and December of 2019, in a vulnerable region of the city of São Paulo, Brazil. Families covered by three public primary healthcare units represented the selected clusters. After study inclusion, participants were asked about signs and symptoms related to COVID-19, and had collected 10 mL of blood for serology testing. A total of 272 individuals from 185 families were included in the study, out of the 400 eligible individuals for inclusion, resulting in a non-response rate of 32%. The post stratified prevalence of individuals infected by SARS-CoV-2 was 45.2% (95% CI: 39.4–51.0%), with a proportion of asymptomatic cases of 30.2% (95% CI: 23.3–38.0%). This population-based serosurvey identified a greater prevalence of infected individuals by SARS-CoV-2 compared to data from the beginning of the pandemic, and from a recent citywide serosurvey, with a similar proportion of asymptomatic individuals. It demonstrated the value of primary healthcare services for disease surveillance activities, and the importance of more focused serosurveys, especially in vulnerable locations, and the need to evaluate new surveillance strategies to take into account asymptomatic cases.

## Introduction

Coronavirus disease 2019 (COVID-19), caused by the severe acute respiratory syndrome coronavirus 2 (SARS-CoV-2), was first identified in December of 2019 following the reporting of a cluster of atypical pneumonia cases in Wuhan, China. Within months it had spread to almost all nations of the world, being declared a pandemic by March 11, 2020 by the World Health Organization (WHO) [1], and causing more than 2.5 million deaths worldwide, until March of 2021 [2]. Brazil recorded the first case of SARS-CoV-2 on February 26, 2020, and virus transmission evolved from imported cases only to local and finally community transmission very rapidly, with the federal government declaring nationwide community transmission on March 20, 2020 [3]. As of March of 2020, Brazil has reported the largest number of cases in Latin America, with more than 10 million cases and 250 thousand deaths [4], with the city of São Paulo, the largest in the country with approximately 12 million people, as the epicenter of the pandemic with more than 600 thousand confirmed cases and 18 thousand deaths [5, 6].

Many countries, including Brazil, implemented physical distancing, quarantine, lockdowns, and travel restrictions to control the epidemic. In many places, these measures successfully contained the initial wave, however with the flexibilization of restrictions countries experienced a second wave of infections, demonstrating how difficult it is to achieve sustained control of the disease [7]. The diverse clinical presentation of COVID-19, including asymptomatic infection, mild, severe and life-threatening illness [8], combined with the possibility of transmission from asymptomatic individuals, make it difficult for public health strategies currently deployed to be successful at controlling the spread of the virus [9].

Even with vaccination underway in many countries, there is still uncertainty regarding the control of the disease, given the emergence of COVID-19 variants that could elude immune responses [10], and the challenges of developing these new vaccines, and of producing and distributing billions of vaccine doses [11, 12]. Therefore, it remains relevant to keep improving our understanding of the distribution of COVID-19, especially of asymptomatic individuals, among different populations to inform public health strategies. In addition, evidence showing racial, economic, and health inequalities among individuals infected by and dying from COVID-19 [13, 14] demonstrates the importance of obtaining this type of data from more vulnerable populations.

Hence, this population-based serosurvey had the objective of estimating the prevalence of individuals 18 years of age and older infected by SARS-CoV-2, and the proportion of asymptomatic individuals, among a vulnerable population living in an urban setting.

## Methods

This was a cross-sectional, single-stage cluster sampling serosurvey conducted between September and December of 2019. The study was approved by the São Paulo Municipal Health Department ethics committee (CAAE 33629020.8.3002.0086) and by the Hospital Israelita Albert Einstein ethics committee (CAAE 33629020.8.0000.0071).

### Population and setting

The source population was composed by approximately 41 thousand individuals, 18 years of age or older, registered at one of three public primary health care (PHC) units serving the neighborhood of Paraisópolis, an area recognized as especially vulnerable in the Vila Andrade district at the city of São Paulo, Brazil. No other additional inclusion/exclusion criteria were used.

The Brazilian health system is made up of a public-private mix with three interconnected subsectors: the public national health system (Sistema Único de Saúde or SUS), the private (for-profit and nonprofit), and the private health insurance subsectors, individuals can use services in all three [15]. All publicly financed health services and most common medications are universally accessible and free of charge for all citizens. Since 1994 the Family Health Program (now called the Family Health Strategy, or FHS) has reorganized PHC in the public sector [16]. Expansion of SUS has enabled a continuous increase in FHS coverage, from 7.8% to 58.5% between 2000 and 2016, but still with great variability between states, ranging from close to 50% in the Southeast region to close to 80% in the Northeast region [17].

The three public PHC facilities included in the study are responsible for an area with approximately 70 thousand individuals [18], with 85.8% of this total population covered by one of 18 FHS teams. Each FHS team is composed of a physician, a nurse, two nurse assistants, and five community health workers that are organized geographically, covering populations of up to 1 thousand households, with no overlap or gap between catchment areas [16]. Although the state of São Paulo has the highest development index in the country, there is significant variability among and within cities. The city of São Paulo is no exception, as shown by the distribution of its districts’ values for the GeoSES, a composite index summarizing the main dimensions of the Brazilian socioeconomic context and which ranges from -1 (worst) to 1 (best) [19]. The median value for São Paulo’s districts is -0.13, ranging from -0.85 to 0.91, with Paraisópolis presenting a value of -0.56 [20].

### Study procedures

Survey teams visited each selected family and eligible members were invited to participate in the study, given they provided written informed consent. After study inclusion, participants were asked about signs and symptoms related to COVID-19, and provided 10 mL of blood for serology testing. If the first contact with a family or family member was unsuccessful, up to five subsequent attempts were made. Blood samples could be collected up to two days after informed consent was obtained, and family members could be recruited on separate days.

### COVID-19 symptoms questionnaire

Participants were questioned about the occurrence of the following symptoms [21] from March 1, 2020, until the day of the study visit: measured (temperature ≥37.8°C) or perceived fever, dry or productive cough, tiredness, sore throat, difficulty swallowing, difficulty breathing or shortness of breath, muscle pain, headache, diarrhea, conjunctivitis, nasal congestion, loss of smell and/or taste, skin rash, and discoloration of the fingers or toes. Symptoms were collected in paper-based forms. The data were entered a single time by two investigators into REDCap (Research Electronic Data Capture), a secure, web-based software platform designed to support data capture for research studies [22]. Participants that were symptomatic at study inclusion were referred to their reference FHS teams to be evaluated and managed in accordance to the current COVID-19 clinical management guidelines established by the Municipal Health Department of the city of São Paulo [23].

### Serology testing

The blood samples were collected by nurse assistants with serum separator tubes, and were transported within 4 hours of collection to the clinical laboratory of Hospital Israelita Albert Einstein. Samples were centrifuged at 3000 g for 15 minutes to separate the serum. Serum samples were analyzed to detect total antibodies against SARS-CoV-2 using an immunoassay, Elecsys® (Roche Diagnostics), acquired at COBAS 8000, following the manufacturer’s instructions, and were labeled reactive (cut off ≥1.0) or non-reactive (cut off <1.0), as recommended. Participants received the results of their SARS-CoV-2 serology through their reference FHS team.

### Sample size and statistical analysis

Taking into account the worldwide uncertainty about the prevalence of infected individual by SARS-CoV-2 at the time of the study design [24], a value of 25% was selected for the sample size calculation, in addition to a margin of error of 6%, considered acceptable to serosurveys of other airborne infectious diseases [25], a 95% confidence interval (CI), a design effect of 1.5, and a non-response rate of 25%. The clusters were individual families registered at one of the three PHC units included in the study, selected by simple random sampling from the patient registries. The final sample size had 185 families representing 400 individuals 18 years of age or older, and household rosters were not updated during the survey. Patient and family registries are maintained by community health workers, that have the responsibility of including every family and individual in her coverage area. Information is updated through periodic household visits for registered families and individuals, and registration is offered to non-registered families and individuals. The information gathered is imputed through tablets into a web-based application.

The seroprevalence of antibodies to SARS-CoV-2, and the proportion of individuals with a reactive test who were asymptomatic, were calculated alongside their respective 95% CIs, taking into account the study design with the package “survey” from R [26]. Poststratification by sex and age groups (18–24 years of age, then five-year intervals, and ≥100 years of age) was used to adjust these estimates for non-response. The post stratified prevalence of individuals infected by SARS-CoV-2, and of asymptomatic individual, stratified by sex and age (18–29, 30–49, and ≥50 years of age) were also calculated.

All analyses were performed with the R software environment for statistical computing and graphics version 4.0.3 [27] and Python version 3.7.4 [28].

## Results

A total of 272 individuals from 185 families were included in the study, out of the 400 eligible individuals for inclusion, resulting in a non-response rate of 32%. All 272 had serology results, and 269 responded to the symptoms questionnaire.

The study population included more female and fewer male participants, who were also slightly older than the source population (Table 1).

**Table 1:** Distribution of sex and age for the source and study populations.

	Study Population	Source Population
	(N = 272)	(N = 41,783)
**Females**		
% (95% CI)	66.5 (61.2–71.0)	54.5
Median age* (p25; p75)	40.1 (30.6; 52.1)	36.0 (26.0; 46.0)
**Males**		
% (95% CI)	33.5 (28.5–39.0)	45.5
Median age* (p25; p75)	37.6 (26.7; 48.7)	35.0 (26.0; 46.0)

CI, confidence interval; p25, 25th percentile; p75, 75th percentile.

*Age in years.

A total of 119 individuals had a positive SARS-CoV-2 serology, resulting in an unadjusted prevalence of 43.8% (95% CI: 37.7%–50.0%). A total of 118 individuals with a positive SARS-CoV-2 serology responded the symptoms questionnaire, with 31 of them being asymptomatic, resulting in a proportion of asymptomatic cases of 26.3% (95% CI: 18.7–36.0%). The post stratified prevalence of individuals infected by SARS-CoV-2 was 45.2% (95% CI: 39.4–51.0%), with 30.2% (95% CI: 23.3–38.0%) of all infections asymptomatic.

The post stratified prevalence of individuals infected by SARS-CoV-2 stratified by sex and age can be found in Table 2 and the related proportion of asymptomatic individuals in Table 3. Statistically significant differences were found among females and the total population older than 50 years old, which presented a significantly higher proportion of asymptomatic individuals when compared to individuals 30 to 49 years of age.

**Table 2:** Prevalence of individuals infected by the SARS–CoV–2, stratified by sex and age.

	N	Females % (95% CI)	N	Males % (95% CI)	N	Total % (95% CI)
**Total**	181	44.6 (38.0–51.0)	91	44.0 (34.5–54.0)	272	45.2 (39.4–51.0)
**18–29 years**	44	49.7 (34.8–65.0)	27	33.9 (18.2–54.0)	71	42.3 (31.1–52.0)
**30–49 years**	82	45.4 (34.9–56.0)	43	52.2 (37.2–67.0)	125	48.4 (39.7–57.0)
**≥50 years**	55	39.3 (28.9–51.0)	21	45.4 (28.1–64.0)	76	41.5 (31.7–52.0)

CI, confidence interval.

**Table 3:** The proportion of individuals infected by the SARS–CoV–2 who were asymptomatic, stratified by sex and age.

	N	Females % (95% CI)	N	Males % (95% CI)	N	Total % (95% CI)
**Total**	78	24.5 (17.2–34.0)	40	37.7 (25.5–52.0)	118*	30.2 (23.3–38.0)
**18–29 years**	22	28.9 (12.7–53.0)	9	20.2 (5.7–52.0)	31	24.8 (12.3–44.0)
**30–49 years**	36	11.7 (4.4–27.0)	23	35.4 (18.0–58.0)	59	22.2 (13.0–35.0)
**≥50 years**	20	39.6 (27.9–53.0)	8	65.9 (38.4–86.0)	28	49.7 (37.4–62.0)

CI, confidence interval.

*One infected individual did not respond the symptoms questionnaire.

All symptomatic participants with a positive serology completed their symptoms questionnaire. Headache, nasal congestion, loss of smell and/or taste, and muscle pain were the four most frequently reported symptoms, with a prevalence above 40% (Table 4).

**Table 4:** Distribution of reported symptoms among symptomatic individuals with positive serology for SARS-CoV-2 (N = 87).

	Total	% (95% CI)
**Headache**	53	60.9 (50.4–70.7)
**Nasal congestion**	44	50.6 (40.2–60.9)
**Loss of smell and/or taste**	43	49.4 (39.1–59.8)
**Muscle pain**	39	44.8 (34.7–55.3)
**Dry or productive cough**	30	34.5 (25.1–44.8)
**Tiredness**	30	34.5 (25.1–44.8)
**Sore throat**	27	31.0 (22.1–41.3)
**Fever**	25	28.7 (20.0–38.8)
**Shortness of breath**	23	26.4 (18.0–36.4)
**Diarrhea**	20	23.0 (15.1–32.6)
**Difficulty swallowing**	17	19.5 (12.3–28.8)
**Conjunctivitis**	6	6.9 (2.9–13.7)
**Skin rash**	5	5.7 (2.2–12.1)
**Discoloration of fingers or toes**	4	4.6 (1.6–10.6)

CI, confidence interval.

## Discussion

This population-based serosurvey took advantage of the existing public PHC structure in Brazil to estimate the prevalence of infection by SARS-CoV-2, and the proportion of asymptomatic cases, among individuals 18 years of age or older living in a vulnerable region of the city of São Paulo.

Since SARS-CoV-2 infection can range from asymptomatic to severe disease, surveillance data based on RT-PCR confirmed cases likely underestimate the real prevalence and spread of the virus, with a significant number of infected individuals presenting subclinical disease not being detected. Therefore, serological screening represents a useful addition to PCR-based diagnosis to fill in the details in the surveillance pyramid, gain insight into the dynamics of specific antibody responses during and after the spread of the virus, and inform health authorities about seroprevalence at any given moment of the epidemic [29, 30].

A recently published systematic review that evaluated the worldwide SARS-CoV-2 seroprevalence, up to August of 2020 [30], found a prevalence of 1.45% (0.95–1.94%) in South America and of 0.96% (0.52–1.40%) in Brazil (based on 15 data sets) for the general population. It did not find differences in prevalence between females and males or in different age strata among adults. Although these results were in accordance to what was expected during the first wave of the pandemic, the authors pointed out that the analyses included a period when the COVID-19 spread had not accelerated in the Southern Hemisphere. The latest results from a population-based serosurvey for SARS-CoV-2 conducted by the São Paulo Municipal Health Department in January of 2021, and that covered the entire city found an overall prevalence of 14.6% among individuals 18 years of age or older [31]. It also identified a higher prevalence in regions with lower values of the Human Development Index, with an overall prevalence of 22% for these locations. This is in accordance with another serosurvey conducted in 133 urban areas in Brazil, that found that individuals in the poorest quintile were 2.16 times more likely to test positive for SARS-CoV-2 than those in the wealthiest quintile, and those with 12 or more years of schooling had a lower prevalence of infection than those with less education [32]. The present study found a much higher prevalence among adults, greater than 40%. This could be closer to the actual prevalence in the source population, since it was based on a larger sample focused on a specific region, in contrast to the smaller samples from many regions used in the municipal wide serosurvey, and could point to the importance of conducting larger localized surveys in vulnerable locations.

Another recently published systematic review [33] found a median of 32.7% (interquartile range, 28.7% to 43.4%) of asymptomatic infections among SARS-CoV-2 positive persons, which is in line with the proportion of 36.1% of asymptomatic infections found by the citywide COVID-19 serosurvey conducted by the São Paulo Municipal Health Department, and with the one found by the present study. These high proportions of asymptomatic individuals represent a challenge for implementing successful surveillance strategies, that have mainly relied on passive identification of symptomatic cases, monitoring and contact tracing. To better control and manage the SARS-CoV-2 pandemic, the effectiveness and cost-effectiveness of active surveillance strategies of the wider population (i.e., to identify and isolate infected individuals regardless of symptoms) should be evaluated in order to guide the improvement of preventive strategies [34, 35]. A systematic review and meta-analysis that evaluated the prevalence of symptoms among adults infected by SARS-CoV-2 found fever (78% [95% CI: 75%–81%]), cough (57% [95% CI: 54%–60%]) and fatigue (31% [95% CI: 27%–35%]) as the most prevalent symptoms [36], which are different from the most frequent symptoms identified by this study, that included headache, nasal congestion, loss of smell and/or taste. The retrospective design of the present study made it vulnerable to recall bias, with study participants asked to accurately recall symptoms that could have occurred even months earlier, in addition referred symptoms could have been to other causes than the COVID-19 infection. However, the awareness of and memory for symptoms related to COVID-19 were probably heightened among the general population during the study period, what could explain the difference in the prevalence of reported symptoms, and also impacted the estimate of the proportion of asymptomatic individuals.

Additional limitations of the present study are the inclusion of adults only, the restriction to a small region within the city of São Paulo, and the resulting limited sample size. However, the area evaluated is likely similar to other vulnerable areas of the city, making it possible to extrapolate the study results, and the sample size was adequate to estimate the population prevalence with an acceptable margin of error.

## Conclusion

This population-based serosurvey in a vulnerable region of the city of São Paulo, Brazil, identified a greater prevalence of infected individuals by SARS-CoV-2 compared to data from the beginning of the pandemic. It also found a greater prevalence of infected individuals, in comparison to a recent citywide serosurvey, but with a similar proportion of asymptomatic individuals. It demonstrated the value of PHC services for disease surveillance activities, the importance of more focused serosurveys, especially in vulnerable locations, and the need to evaluate new surveillance strategies to take into account asymptomatic cases.

## Supporting information

S1 Data (XLSX)
